# IGD: a simple, efficient genotype data format

**DOI:** 10.1093/bioadv/vbaf205

**Published:** 2025-08-26

**Authors:** Drew DeHaas, Xinzhu Wei

**Affiliations:** Department of Computational Biology, Cornell University, Ithaca, NY 14850, United States; Department of Computational Biology, Cornell University, Ithaca, NY 14850, United States

## Abstract

**Motivation:**

While there are a variety of file formats for storing reference-sequence-aligned genotype data, many are complex or inefficient. Programming language support for such formats is often limited. A file format that is simple to understand and implement—yet fast and small—is helpful for research on highly scalable statistical and population genetics methods.

**Results:**

We present the Indexable Genotype Data (IGD) file format, a simple uncompressed binary format that can be more than 100× faster and 3.5× smaller than *vcf.gz* on biobank-scale whole-genome sequence data. The implementation for reading and writing IGD in Python is under 350 lines of code, which reflects the simplicity of the format.

**Availability and implementation:**

A C++ library for reading and writing IGD, and tooling to convert .vcf.gz files, can be found at https://github.com/aprilweilab/picovcf. A Python library is at https://github.com/aprilweilab/pyigd.

## 1 Introduction

Genetic polymorphism data is typically stored in a tabular format that can be thought of as an S×N matrix. The rows represent *S* sites and the columns represent *N* individuals, where each site has at least one alternate allele that differs from the reference sequence, but may have many (a multiallelic site). Variant Call Format (VCF) (Danecek *et al.* 2011) and its compressed form (*.vcf.gz*) are mainstays of tooling that process such tabular genotype data. The VCF format is very flexible, and its plaintext nature makes it easy to understand, construct, and parse. However, it is inefficient to store and process for large-scale datasets, as evidenced by the proliferation of faster and more compact formats. Among alternate formats, BCF (Li 2011), BGEN (Band and Marchini 2018), and BED (Purcell *et al.* 2007) have popular tooling support. Newer, more efficient formats such as PGEN (Rivas and Chang 2024), XSI (Wertenbroek *et al.* 2022), Savvy (LeFaive *et al.* 2021), GTshark (Deorowicz and Danek 2019), GRG (DeHaas *et al.* 2024), Genozip (Lan *et al.* 2021), and bref3 (Browning *et al.* 2018) leverage the similarity between samples at nearby genetic positions [due to linkage-disequilibrium (LD)] to compress genotype data to an impressive extent. By looking at multiple variants together to determine a relationship between them, LD-based methods produce smaller file sizes.

Here we present the Indexable Genotype Data (IGD) format, which is designed by the (sometimes at odds) principles of simplicity and efficiency. IGD encodes tabular genotype data as hard calls, similar to pVCF and BED. The only metadata it stores (optionally) are identifiers for variants and individuals, however, the IGD tooling can export variant-related metadata from VCF as plaintext that can be easily manipulated. IGD is uncompressed, which makes reading and writing the format easy to implement and avoids the need for external compression libraries which may not be easily usable across platforms or programming languages. IGD is a binary format, and supports multiallelic variants, any ploidy up to 255, is contained in a single file, and can be constructed in one pass over the input data. IGD can represent both phased and unphased data, but all data in the file must have the same phasedness. Unlike the methods above that leverage LD during file construction, IGD is constructed by examining a single variant at a time.

IGD is primarily focused on the use cases of statistical and population genetics, especially as an input format for new tools and research prototypes in these areas. Similar to the BIM and FAM files that go along with the plink BED representation (Purcell *et al.* 2007), the IGD tooling keeps most metadata in external plaintext files, and focuses on efficient representation of the genotype data itself. This is most convenient when metadata is only useful for the preanalyses (e.g. data filtering) and postanalyses (e.g. variant prioritization), but it is not directly used in the computation involving genotypes; for the majority of the population and statistical genetic analyses, metadata is not directly used in computation. The IGD data storage is similar to a sparse-matrix representation of the genotype matrix, and it is intended for fast access to the entire dataset, or very large subsets of the data, for example to perform repeated numerical calculations or to process haplotypes for tree- and graph-building algorithms. In contrast, methods like GQT (Layer *et al.* 2016) and BGT (Li 2016) are focused on querying large datasets by variant, sample, metadata, or a combination thereof, to find subsets of interest.

## 2 Methods

Given that we have *N* individuals in a dataset, we number them 0…(N−1). There are $N_{H}=N\times \mathrm{ploidy}$ haploid samples of these individuals, which are similarly numbered $0\ldots (N_{H}-1)$. Throughout when we refer to a “sample” we mean a haploid sample. There are *M* variants in a dataset, each of which can be uniquely identified by the pair (base-pair position, alternate allele), and *Q* variants that contain at least one sample with missing data. There are a few significant aspects of the IGD format worth highlighting.

**All polymorphic sites are stored using biallelic format.** Instead of storing a row per site ($S\times N_{H}$ matrix), IGD stores a row per variant ($M\times N_{H}$ matrix). Multiallelic sites are supported by expanding them into a row per variant. For example, a (*k *+ 1)-allelic site with *k* alternate alleles (without missing data) is expanded into *k* variants that all have the same position and reference allele, but different alternate alleles. The original multiallelic sites can be recovered by aggregating the IGD variants by position, however, keeping the data as an $M\times N_{H}$ matrix is often convenient for statistical genetics or population genetics applications. In practice, IGD is an $(M+Q)\times N_{H}$ matrix, since missing data is encoded as a row of samples representing the ones with missing data, instead of representing those with the alternate allele.

**IGD contains an internal index.** The index contains the genomic position (in base pairs) of each variant, and can be cross-referenced to the genotype data, the allele strings, or variant IDs. By keeping the contents of the index small (16 bytes per variant) we keep the cost of reading it from disk very small. All variant-related data in IGD can be randomly accessed by *i*, the row number of that variant in the IGD index.

**IGD uses one of two compact genotype formats per variant.** Each row of genotype data is represented as the set of samples that have the variant/alternate allele. The two simple, compact ways to store this data are either (i) sparsely as a list of sample numbers or (ii) densely as a bit-vector where each bit at position *i* reflects whether the *i*th sample has the alternate allele (1) or not 0. We can choose between representation (i) and (ii) by examining the allele frequency $p_{i}$ of the variant in question. Sample numbers are represented by 32-bit unsigned integers, which means that if $p_{i}<\frac{N_{H}}{32}$ the sparse representation is more compact (i), otherwise the bit-vector representation (ii) is smaller. It is important to note that a valid IGD file can be constructed using only row representation (i) or only bit vector representation (ii), or any mixture of the two, but the optimally sized IGD will determine which to use on a per-variant basis. Converting between these two representations is trivial, and thus the representation on disk does not have to be the representation used for computation.

### 2.1 File format details

**Figure 1.**
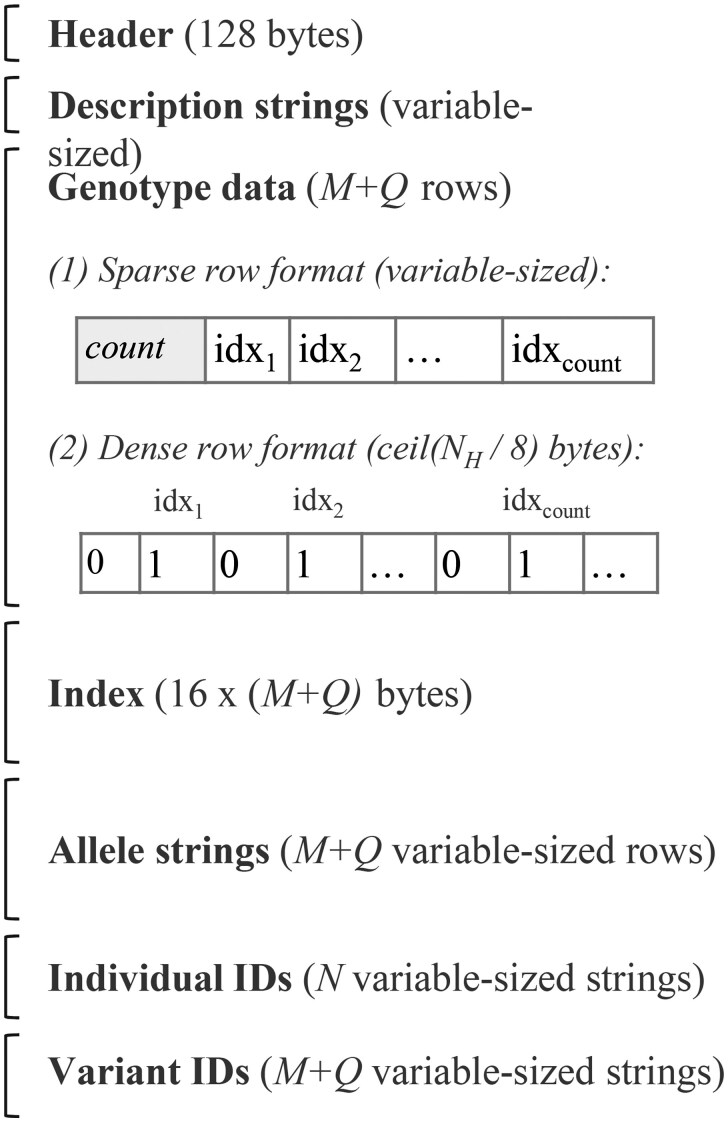
IGD file layout. The layout of an IGD file on disk. *M* is the number of variants, *Q* is the number of variants with missing data, *N* is the number of individuals, and $N_{H}$ is the number of haploid samples. Genotype data rows may be either sparse (a list of haploid sample indexes containing the alternate allele) or dense (a bit-vector with a 1 at an index *i* if the *i*th haploid sample contains the alternate allele).

The layout of an IGD file is shown in Fig. 1. The header contains file offsets for each of the sections after the genotype data, as their positions are unpredictable otherwise, and random access to them is useful. We use the following storage type definitions.

*uint32*: A 32-bit unsigned integer.
*uint64*: A 64-bit unsigned integer.
*string*: A uint32 for the length *k*, followed by *k* bytes for the contents.
*list32*: A uint32 for the length *k*, followed by *k uint32* values for the contents.
*bv*(*w*): A bit-vector of w bits stored at the byte granularity. The number of bytes used is ceil($\frac{w}{8}$). Given a sample index *b* that we want to store as a 1, the byte offset is determined by floor($\frac{b}{8}$). Within that byte we set the (7−(b mod 8))th least significant bit; i.e. if (b mod 8)=0 we will set the most significant bit.

#### 2.1.1 Header

The header is a fixed-size (128 byte) table as described in Table 1.

**Table 1.** IGD header details.

Byte offset	Datatype	Description
0	uint64	Magic number 0x3a0c6fd7945a3481
8	uint64	File format version
16	uint32	Ploidy
20	uint32	Sparsity threshold
24	uint64	Number of variants
32	uint32	Number of individuals *N*
36	uint32	Reserved for future use
40	uint64	64 bits of flags
48	uint64	File offset of the **Index**^1^
56	uint64	File offset of the **Allele strings**
64	uint64	File offset of the **Individual IDs**
72	uint64	File offset of the **Variant IDs**
80	48 bytes	Reserved for future use

1 Bold text indicates the referenced file section is described further in the text.

#### 2.1.2 Flags

The least-significant bit of the flags (in the header) signifies phasedness, where a value of 1 indicates phased data.

#### 2.1.3 Description strings

Immediately following the header are two string values. The first is a string describing how the file was created (e.g. “converted from foo.vcf.gz”) and the second is a generic description field.

#### 2.1.4 Genotype data

Immediately following the description strings is M+Q rows of genotype data, where each row is either a *list32* or a *bv*($N_{H}$). The Index (described next) has a flag that indicates the type of each row.

#### 2.1.5 Index

The Index is M+Q rows of 16 bytes each, and can be viewed as two *uint64* values. The first *uint64* value contains the base-pair position associated with the variant in the least significant 48 bits, followed by an 8-bit unsigned integer *numCopies*, and finally bitwise flags in the most significant 8 bits. The currently defined flags are:

**SPARSE=0x01**: If this flag is set the corresponding genotype row is a ***list32***, otherwise it is a ***bv(***$N_{H}$***)***.
**IS_MISSING=0x02**: If this flag is set the corresponding genotype row’s sample list represents missing data. The list of samples in the row do not have a variant call for that polymorphic site.

The *numCopies* value is 0 for phased data, but is 1≤numCopies≤ploidy for unphased data. The second *uint64* value in the Index row contains the file offset of the genotype row for the current variant. The *i*th variant can be randomly accessed directly at IndexStart+(16×i).

#### 2.1.6 Allele strings

This section is M+Q rows, each row contains first the *string* for the reference allele and then the *string* for the alternate allele.

#### 2.1.7 Individual IDs

This section is a *uint64* for the number of strings, followed by that many strings, where the *k*th is the identifier for individual *k*. This section is only present in the file if the corresponding header file offset entry is non-zero.

#### 2.1.8 Variant IDs

This section is a *uint64* for the number of strings, followed by that many strings, where the *i*th one is the variant identifier for the *i*th variant. This section is only present in the file if the corresponding header file offset entry is non-zero.

### 2.2 Phasedness

Unphased data is stored by clearing the phased flag in the header, and storing separate variants for each number of copies of each alternate allele. The *numCopies* value in the index (see above) indicates the zygosity of the currently stored sample list. Additionally, instead of storing haploid sample lists, IGD stores individual-based sample lists for unphased data. That is, it represents an (M+Q)×N matrix (instead of $N_{H}$).

### 2.3 Access patterns

If the variant index *i* is known, we can seek directly to it in the Index and then seek directly to the genotype data for that variant. If any of the string data is needed (alleles, individual IDs, variant IDs) those tables will need to be loaded into memory, so that they are indexable by *i*, or just scanned on disk to find the *i*th entry.

If only a subset of the variants are needed, e.g. based on a region of the genome, the IGD index can be binary searched by variant base pair position. Alternatively, traversing an IGD file is done by seeking to the start of the Index. Starting at variant i=0, each row of the Index is read and if the base-pair position is of interest then the genotype data is accessed using the file offset found in the current (*i*th) row of the Index. String data can be read into RAM one time, or a file pointer can be maintained to the current entry for each string table and incremented whenever *i* is incremented.

Samples and metadata are not indexed in IGD, and therefore cannot be queried efficiently.

## 3 Results

**Figure 2.**
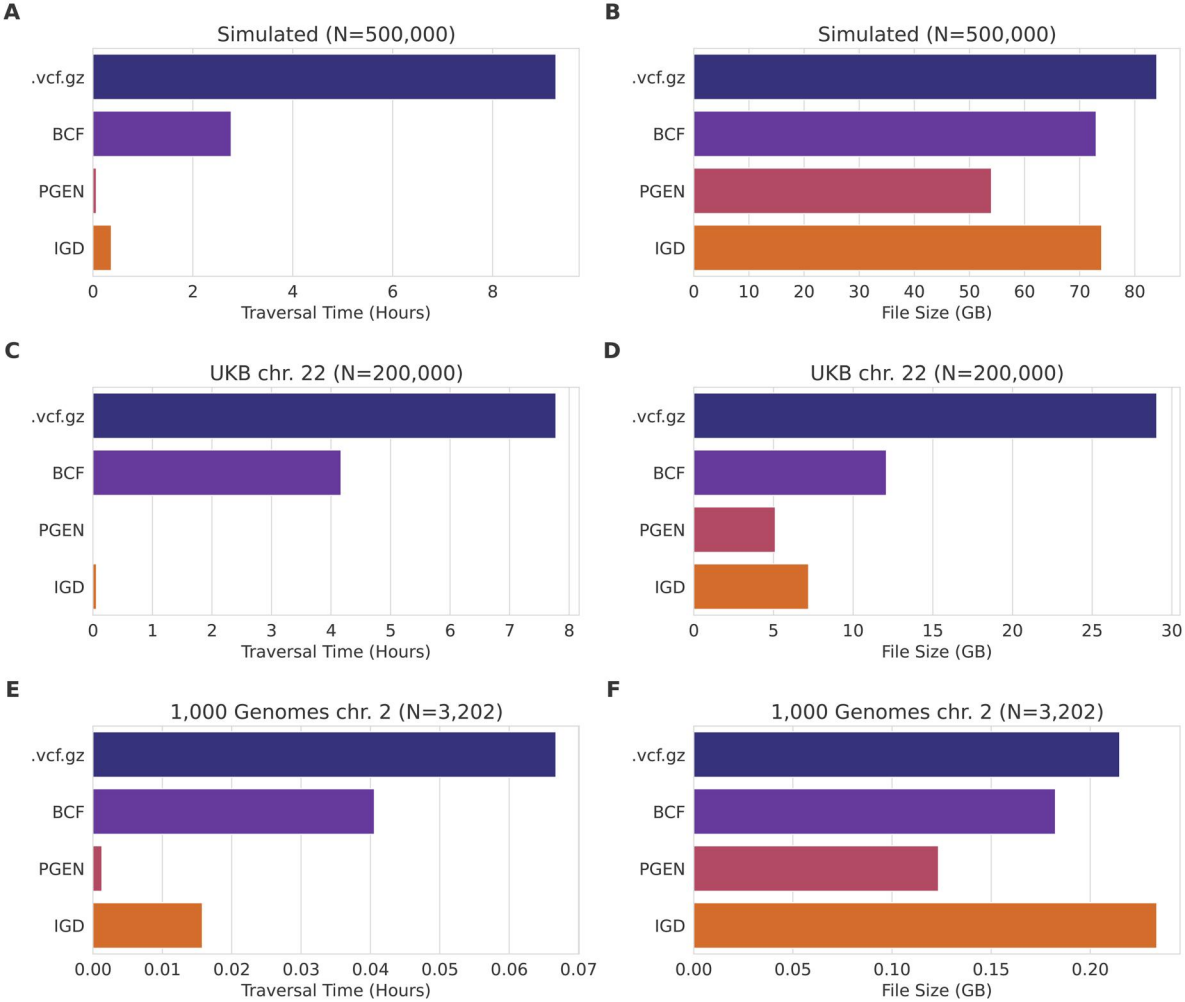
Comparison of file formats. The left panels (A, C, E) show time to traverse the file for datasets of decreasing size, top to bottom: simulated data, UK Biobank WGS data, and 1000 Genomes Project data. The right panels (B, D, F) show the file sizes for the same datasets. Each dataset contains *N* diploid individuals, as labeled in the figure.

We compared file size and data traversal time between IGD, *.vcf.gz*, BCF, and PGEN (Fig. 2). These formats were chosen for their apparent popularity as well as for capturing a spectrum from simple and inefficient (*.vcf.gz*) to more complex, yet very efficient (PGEN). PGEN is on the more complex side because it uses LD-based compression, and also supports eight different storage modes, some of which are for backwards compatibility (Chang 2024).

Allele frequency calculation was used for traversal time, as it is a trivial calculation over all the data in the file. For formats that encode an allele count, such as BCF, PGEN, and IGD (for sparse variants), we do not use that count but read the full sample data for each variant in order to measure the data traversal overhead. plink2 (Purcell and Chang 2024) was used for conversion to BCF and PGEN formats, as well as for allele frequency calculation for *.vcf.gz* and BCF. The API *PgrGetDifflistOrGenovec()* was used for calculating PGEN allele frequency. The BCF files were stripped of additional metadata, and only contained variants and genotypes. All variants (the entire file) are traversed for all comparisons, and tools/code based on C/C++ were used for conversion and traversal of all file types. All conversions and traversals were done single-threaded, except that plink appears to always use at least two threads, so we measured the user + system time (instead of elapsed) for plink, to compensate for this.

Unless otherwise stated, the simulated data was generated via stdpopsim (Adrion *et al.* 2020) and msprime (Kelleher *et al.* 2016), using an out-of-Africa demographic model (Jouganous *et al.* 2017) and sampling 500 000 European individuals, in an attempt to generate data similar to the UK Biobank whole-genome sequence data (Hofmeister *et al.* 2023).

File sizes are all fairly similar on the simulated dataset, ranging from 54 GB (PGEN) to 84 GB (*.vcf.gz*), with IGD and BCF being similarly sized at 73–74 GB. PGEN and IGD are the two smallest formats on the UKB dataset, which is much richer in low-frequency variants [96% variants are MAF <0.1% (Hofmeister *et al.* 2023)] than our simulated dataset (73% of variants are MAF <0.1%), and thus more compactly stored with a sparse representation. IGD is the largest of the compared formats on the data from 1000 Genomes Project (Byrska-Bishop *et al.* 2022). On small datasets like 1000 Genomes, which contain only a few thousand samples, existing formats like VCF and BCF are sufficient, though IGD provides faster traversal. BCF and *.vcf.gz* rely heavily on standard compression algorithms, which is illustrated by the traversal times for IGD and PGEN being faster on larger datasets, especially on the UKB data. PGEN is the smallest and fastest file format, about 30% smaller and 5× faster than IGD.

File format conversion times are summarized in Table 2. PGEN is again fastest, likely partially due to plink’s highly optimized *.vcf.gz* read functionality.

**Table 2.** Conversion times from*. vcf.gz* on large datasets.

Dataset	File Format	Conversion time (h)
UKB chr. 22 (*N* = 200 000)	BCF	32.5
UKB chr. 22 (*N* = 200 000)	PGEN	21.5
UKB chr. 22 (*N* = 200 000)	IGD	21.3
Simulated (*N* = 500 000)	BCF	29.9
Simulated (*N* = 500 000)	PGEN	8.1
Simulated (*N* = 500 000)	IGD	23.6

**Figure 3.**
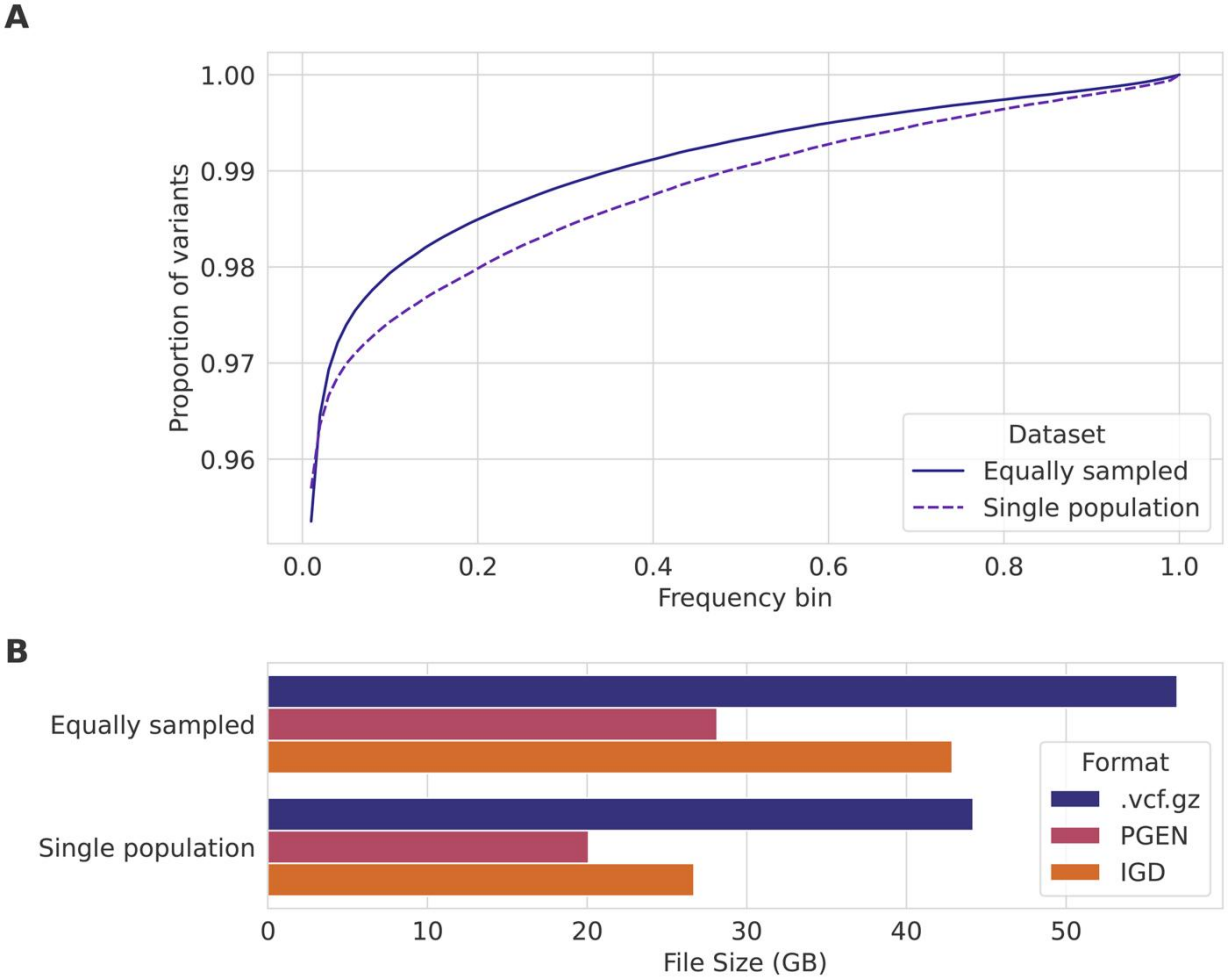
Comparing properties of datasets with samples from a single population or four different populations. The cumulative proportion of variants in each frequency bin, for 100 uniformly sized bins on two simulated datasets using the same demographic model (A), and the corresponding file sizes for different formats (B).

We also investigated how much the demographic composition of the sample set affects the scaling factor. We simulated two datasets of 200 000 individuals each, using the same four-population demographic model as above. The first dataset sampled equally from the four populations (YRI, CEU, CHB, and JPT), and the second dataset sampled only from JPT. The equally sampled dataset has 45% more variants than the single population dataset, which resulted in an increase in file size of 29% for .vcf.gz, 40% for PGEN, and 61% for IGD. The single population (more homogeneous) dataset has a higher *proportion* of very low frequency variants (alternate allele frequency ≤0.01) which is likely why IGD increases in size slightly disproportionately to the number of additional variants in the equally sampled dataset (Fig. 3).

## 4 Discussion

**Figure 4.**
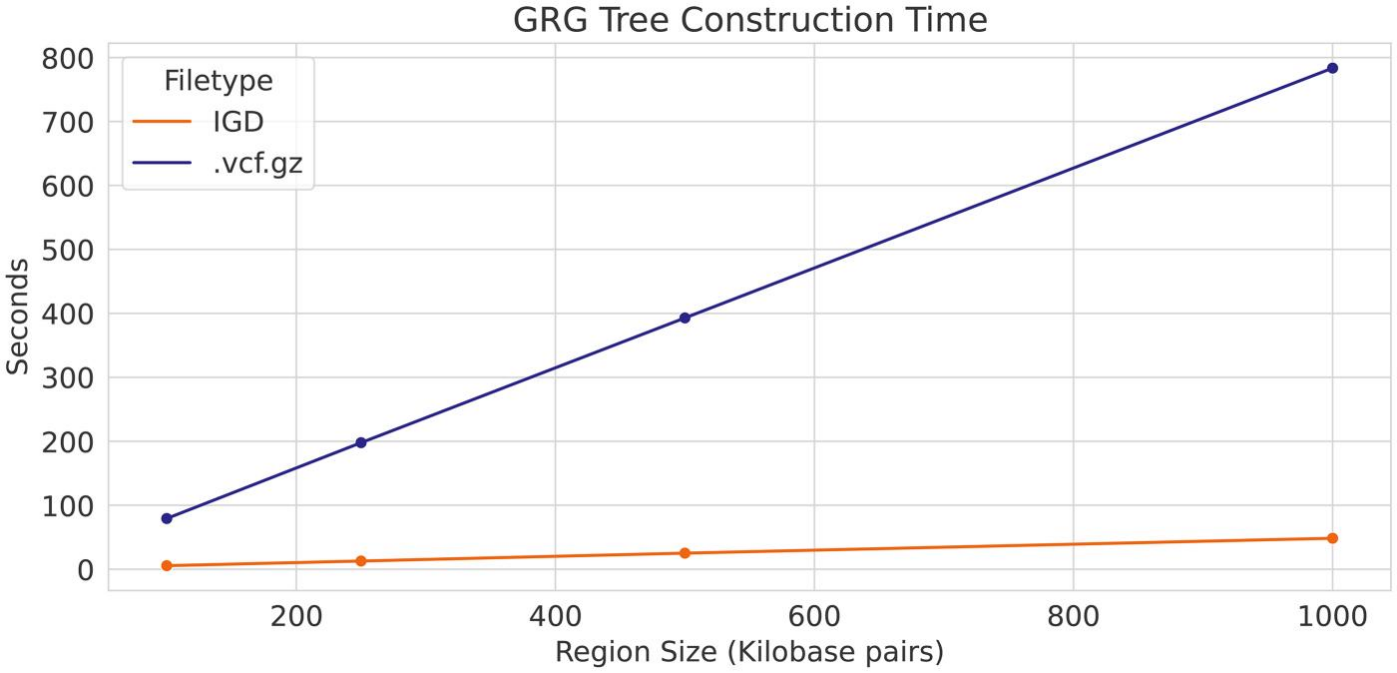
File format impact on GRG tree construction. Genotype Representation Graph (GRG) tree construction time for.*vcf.gz* vs. IGD file formats, for small regions of the genome (*x*-axis) from a simulated dataset with 1 million haploid samples from a homogeneous population. The time of GRG tree construction is estimated using the same region (for varying lengths, on the *x*-axis) from a simulated dataset with 1 million haploid samples.

While some other formats feature even smaller file sizes, the compactness and simplicity of IGD format make it easily usable in bioinformatic tool development. IGD has been successfully used as the input to Genotype Representation Graph (GRG) construction (DeHaas *et al.* 2024), and is essential for the efficiency of that process on biobank-scale data. GRG construction requires fast indexing of genomic regions as well as fast genotype data access. Indexing compressed formats (such as *.vcf.gz* and BCF) can be complex, and creates a separate file for the index. For IGD the index is a fundamental part of the file format, and the time to construct a GRG tree, the first part of GRG construction, is 13× to 15× faster for IGD than for *.vcf.gz* (Fig. 4).

While the primary access mode for IGD is to traverse large parts of the file (e.g. considering it as a genotype matrix), looking up a specific variant position via binary search is fast, 0.08 milliseconds on average (using the Python library, 10 000 random queries, and the simulated dataset described above). All variants are stored in ascending position order, the same as VCF, and accessing all samples at a variant site can be done in linear time by collecting samples in a list for every consecutive variant until the position changes, then a new list is started. The Python library for IGD provides additional useful functionality, such as traversal by site (instead of variant), retrieval of the sample list by variant or by site, and easy modification of an existing IGD file. The C++ library for IGD is implemented as a single header file to make tool integrations easy, and also includes VCF parsing and conversion functionality.

IGD has two different ways to store unencoded, uncompressed data. More compact formats exist, but often have more complexity in how they store data [e.g. PGEN has eight different storage layouts, SNPack has five (Sambo *et al.* 2014)] or how the file is constructed or modified (e.g. analyzing blocks of variants together, calculating patterns of LD and then representing variants in terms of differences to other variants). IGD provides an extremely simple, yet efficient, alternative that focuses on genotype data storage, and ease-of-use for developers of scalable population and statistical genetics prototypes and tools.

## Data Availability

New data in this study were generated through simulations. Simulation model and parameter were fully presented in the manuscript.
